# Role of interleukin 6 polymorphism and inflammatory markers in outcome of pediatric Covid- 19 patients

**DOI:** 10.1186/s12887-024-05071-9

**Published:** 2024-10-01

**Authors:** Reem A. AbdelAziz, Samir Tamer Abd-Allah, Hend M. Moness, Ahmed M. Anwar, Zamzam Hassan Mohamed

**Affiliations:** 1 Pediatric Department, Faculty of Medicine, Minia University, Minia, Egypt; 2 Clinical Pathology Department, Faculty of Medicine, Minia University, Minia, Egypt

**Keywords:** Covid-19, IL-6 polymorphism, Children, Prediction, Severity, Prognostic value

## Abstract

**Background:**

IL-6 polymorphisms were associated to viral infection outcomes through affection of IL-6 production and it is an early indicator of tissue injury and systemic inflammatory response. The study aimed to determine whether genetic IL-6 polymorphisms, serum interleukin-6 level and inflammatory markers (Presepsin, CXCL-10, C3, and C4) are associated with the prediction of disease severity in pediatric COVID-19 patients and its possible use as a prognostic tool in pediatric patients admitted to hospital.

**Methods:**

This prospective cohort study was conducted on 150 children with COVID-19. Patients were divided according to the severity of infection into four groups: group I (mild) 67 cases; group II (moderate) 53 cases, group III (severe) 17 cases and group IV (critical) 14 cases. Serum Interleukin 6, CXCL-10, Presepsin, renal and liver functions, electrolytes, C3, C4, ferritin, and D dimer serum levels were assessed in all patients. The Kruskal Wallis test used to compare parametric quantitative data between studied groups and Mann Whitney test for each pair of groups. Non-parametric quantitative data was compared between studied groups using a one-way ANOVA test and post-hoc Bonferroni analysis for each pair of groups.

**Results:**

Group I: 35 males and 32 females with a median age of 16 months. Group II: 17 males and 35 females with a median age of 13 months. Group III: 6 males and 11 females with a median age of 12 months and group IV: 3 males and 11 females with a median age of 12 months. There was no statistical difference between the studied groups regarding gender and age. Serum levels of IL- 6, serum ferritin; D-dimer, Presepsin and CXCL 10 were significantly higher in both severe and critical groups than the other 2 groups (mild and moderate). ROC curve analysis showed that interleukin-6 and Presepsin were good markers for prediction of severity of COVID-19 among the diseased children. For severe cases, the sensitivity of interleukin-6 was 76.47% and specificity was 92.31%. For critical cases, the sensitivity of interleukin-6 was 71.43% and specificity was 82.35%. The sensitivity of Presepsin was 76.47% and specificity was 88.46% in severe cases. For critical cases, the sensitivity of Presepsin was 78.57% and specificity of 91.2%. There was significant difference in IL-6 572 allelic among moderate cases with the most frequent 42.3% for genotype (GC) and allelic among severe cases with the most frequent 47.1% for genotype (GC). Significant difference in IL-6 174 allelic among critical cases with the most frequent 78.6% for genotype (CC).

**Conclusions:**

Children whom expressed GC genotypes of IL6 (-572G > C) polymorphism are at a considerably higher risk of developing a severe disease. This risk is significantly larger in the severe group of children than in children in critical condition who have GC genotypes of IL6 (-174 G > C) polymorphism. While IL6 (-597G > A) polymorphism has no role in COVID 19 severity in children.

## Background

A new coronavirus was determined in Wuhan, China, around the end of 2019 and named as COVID-19 (Coronavirus Disease 2019). This virus led to outbreak and WHO reported it as a major health problem [1, 2]. Many patients affected by COVID-19 represented by mild to moderate symptoms, but about more than 20% of them were manifested by severe manifestations which lead to respiratory distress, thrombosis or even death [3]. At July 2022, WHO reported that affected children under the age of 5 years and children their age from 5–14 years were 2.47% and 10.44% respectively. It was noticed that COVID-19 infection usually associated with severe inflammatory responses that accompanied by release of excess amount of pro-inflammatory cytokines (interferon gamma (IFN-y), tumor necrosis factor alpha (TNF-α), interleukin-6 (IL-6), IL-10, IL- 18, in addition to inflammatory markers (CXCL-10, presepsin, C3 and C4). This condition is called cytokine storm [4]. Various researches have been done to know mechanism of COVID-19 infection, but its real pathophysiology is not fully clear. It is established that cytokine storm is responsible for disease severity ranging from hospitalization to mortality state [5, 6].

Despite that there are several factors associated with COVID 19 severity, as age, sex, BMI (body mass index) …etc., severe course of disease was noticed in individuals without any risk conditions. So heterogonous genetic factors may have a role in the disease severity Recognizing the single nucleotide polymorphisms (SNPs) present in cytokine and chemokine genes helping to understand cytokine storm in COVID-19 [7, 8].

Numerous studies have stated that IL-6 measure in serum have a predictive value for COVID-19 disease severity. IL-6 found to be superior to ordinary marker such as CRP, fibrinogen, D-dimer, lymphopenia in detection of severity of COVID-19 infection [9–11]. Multiple systematic reviews and meta-analyses consider IL-6 a useful marker for disease severity and mortality prediction. They also mentioned IL-6 is useful in therapeutic response monitoring [12].

IL-6 polymorphisms were associated to viral infection outcomes through affection of IL-6 production [13]. The gene responsible for IL-6 present in chromosome 7p21- 14, there are many single-nucleotide polymorphisms (SNPs) in the gene's coding and non-coding areas was described. The variation of IL-6 level among individuals may be related to SNPs that take place in regulatory regions, which will influence the IL-6 blood level [14]. Multiple studies showed that variations in the IL-6 gene promoter at rs1800796 (572 G > C), rs1800795 (174 G > C) and rs1800797 (597 G > A) are linked to IL-6 levels in serum as well as the occurrence, prevalence and development of several diseases including sepsis, chronic obstructive pulmonary disease, and hepatocellular carcinoma [15, 16]. The role of polymorphisms in genes encoding IL-6 in the severity of COVID-19 is unclear. The effect of these polymorphisms on disease severity and clinical outcomes has been assessed in different populations. There is limited data about the role of IL-6 polymorphisms in pediatric COVID-19 and genetic background has been frequently ignored to be eventually confirmed as a major player in COVID-19 infection [17, 18].

Up to our knowledge, our study is the first one to evaluate the role of genetic IL-6 polymorphisms in the prediction of disease severity and clinical outcomes in Egyptian pediatric COVID-19 patients.

Presepsin is a soluble CD14 subclass which is considered as a marker for patients with sepsis, many studies report its role in detecting sepsis, its severity and outcome [19]. Moreover, high Presepsin level can be used as an important biomarker in assessment of COVID-19 patients [20, 21].

Chemokine ligand 10 (CXCL10) is a CXC chemokine released in reaction to interferon gamma. It is important for the activation of natural killer cells, neutrophils and lymphocytes. CXCL10 has an important role in the advance of the disease and severity of inflammation caused by COVID 19 [22, 23].

Complement system has role in protection against different infectious agent, through activation of classical or alternative pathways [24]. Measurement of C3 and C4 is used to determine and evaluate complement activation [25].

Our study aimed to determine whether genetic IL-6 polymorphisms, serum interleukin-6 level and inflammatory markers (Presepsin, CXCL-10, C3, C4) are associated in the prediction of disease severity in pediatric COVID-19 patients and its possible use as a prognostic tool in pediatric COVID-19 patients admitted to hospital.

## Methods

The study aimed to measure different inflammatory cytokines, serum interleukin-6, CXCL-10, presepsin, C3, C4 and IL-6 SNP 174, 574, 597 and correlates their levels with clinical signs, disease severity and patients' outcome. This was a prospective cohort study; our patients were selected from the pediatrics inpatient department and outpatient pediatric clinic at Minia University's Faculty of Medicine between March 2021 and June 2021. Both verbal and written consents were obtained from all patients' parents. The study was approved from Minia College of Medicine Ethical Committee according to the Helsinki Declaration and its modifications. Ethical approval number 35: 2021.

One hundred and fifty patients were involved in our study. All children with suspected COVID-19 were confirmed by positive PCR nasal swab for SARS-CoV-2 [26, 27]. Our patients were divided according to the severity of infection based on WHO criteria [28] into four groups: group I (mild): 67 cases presented with suspected COVID-19 symptoms (e.g., fever headache, sore throats, coughing, lethargy, muscle aches, loss of taste, smell, nausea, vomiting, and diarrhea) but they lack dyspnea, shortness of breath and abnormal chest radiograph, group II (moderate): included 53 cases presented with mild symptoms together with tachypnea and/or + ve COVID-19 specific imaging but with normal oxygen saturation (SpO2 ≥ 92%). Group III (severe): 17 cases presented with tachypnea and other signs of respiratory distress, SpO2 < 92%, PaO2/FiO2 < 300, lung infiltrates > 50% of lung image or progressive lung lesion within 24–48 h. Group IV (critical): included 14 cases presented by: acute respiratory distress syndrome (e.g. tachypnea, PaO2/FiO2 ratio < 200 despite oxygen therapy or SpO2 < 92%) and/ or multi-organ dysfunction and/or septic shock or coma.

All children with moderate, severe, and critical presentations (Groups II, III, and IV) were admitted at quarantine sector in our intensive care unit.

We excluded any case with chronic disease as chronic liver diseases, endocrine disorders (diabetes mellitus, hypo, hyperthyroidism, adrenal diseases, etc.), Crohn's disease and.

other associated growth failure, hematological diseases (thalassemia, sickle cell disease), collagen disease (systemic lupus erythematosus, juvenile idiopathic arthritis, and others), metabolic, cardiac disease such as (congenital heart disease, coronary artery disease, hypertension, etc.), chronic chest disease (bronchial asthma, other obstructive or restrictive lung disease), and chronic drug intake. Any case refused to participate and ages less than 1 month and more than 18 years were not included in this study.

All of the patients were subjected to (a) clinical history: a comprehensive medical history was taken considering age, sex and any associated diseases or drug intake, (b) clinical examination: anthropometric measures, including weight, height and body mass index, measurement of blood pressure, pulse, temperature, respiratory rate. Chest, heart, and abdominal examinations and (c) laboratory investigations which involved routine laboratory investigation as Complete Blood Count (CBC), D-dimer, liver and kidney functions, electrolytes, serum ferritin level, C-reactive protein (CRP) and special laboratory investigations Serum interleukin 6, Complement 3 (C3), Complement 4 (C4), CXCL 10, Serum Presepsin and SNPs of IL-6 (174 G / C), (572 G /C), and (597 G / A).

About 7 ml of venous blood was taken from each individual by sterile venipuncture. This sample was split up as follows: one milliliter was taken and placed in a sterile vacutainers tube containing EDTA solutions for CBC assay, one ml was collected on vacutainers tube containing EDTA solutions tube for IL-6 SNPs detection, 0.9 ml of blood on a tube containing 0.1 ml tri sodium citrate for measuring D-dimer and 4 ml of venous blood were placed in separator gel serum tubes, samples were incubated for thirty minutes at 37 °C before centrifugation for fifteen minutes at 3,500 rpm. Then serum was used to assay renal, liver functions, electrolytes, CRP, serum ferritin. The residual serum was kept at -20 °C for assay of special investigation.

A- Routine investigations: CBC was done by using automated cell counter Sysmex XN-1000TM hematology autoanalyzer (Japan's Sysmex, Kobe). Renal function, liver function, electrolyte, C3, C4 and serum ferritin assayed by automated chemical analyzer Mindray BS-800, China. CRP level was measured by GENIUS PA54 Specific Protein Analyzer, Chain. D dimer was determined by using the automated immunoassay quantitative Enzyme Linked Fluorescent Assay (ELFA) technique by (Mini Vidas, Biomerieux, France).

B- Special investigations:

CXCL-10: was assayed by ELISA, Kit was supplied by (abcam, catalog no. ab83700).

Presepsin: Kit was provided by (BT- Bioassay technology laboratory) catalog number E3754Hu.

Serum Interleukin 6: Kit was provided by (BT- Bioassay technology laboratory) catalog number E0090Hu.

IL-6 SNPS by PCR- REFLP (polymerase chain reaction-restriction fragment length polymorphism): was assayed through two steps:

1- DNA extraction: from two mL EDTA-anticoagulated peripheral blood DNA was extracted and kept at -40 °C until genotyping analysis (Qiagen, Germany). 2- Using the polymerase chain reaction-restriction fragment length polymorphism technique, three SNPs, rs1800795 (174 G / C), rs 1,800,796 (572 G / C), and rs1800797 (597 G/A) were genotyped. DNA fragments were amplified in a 15μL reaction mix made from 0.5 L extracted DNA, 1 μL of each primer, 7 μL master mix, and 5.5 μL distilled water. An initial denaturation step of 95 °C for three minutes, followed by 30 cycles of denaturation at 94 °C for 45 s, an annealing temperature of 30 s at 58 °C for rs1800795 and 20 s at 61 °C for rs1800796 and rs1800797 and a final extension step at 72 °C for ten minutes were the PCR cycle for the three SNPs. Each SNP has specific RFLP digestion enzyme (restriction enzyme) ( according manufacture instruction kit was supplied by Thermo Fisher Scientific Inc). Taql restriction enzyme was used to digest rs 1800795 (174 G/C), Bsrbl restriction enzyme was utilized for rs1800796 (572 G/C) lastly Btscl restriction enzyme was used for rs1800797 (597 G/ A) [29].

### Statistical analysis

- The data were analyzed using SPSS version 22 (Statistical Software Package version 22). There was a descriptive analysis done. To illustrate quantitative data, the mean, standard deviation, and range were utilized Qualitative data were expressed as frequencies and percentages and compared across groups using the Chi square test. SPSS version 22 was used to make the graphs. A *P* value was considered significant if it was less than 0.05. Non-parametric quantitative data was compared between the four groups using The Kruskal Wallis test, and for each pair of groups, the Mann Whitney test was utilized. For parametric quantitative data comparing the four groups, a one-way ANOVA test was used. For each pair of groups, post-hoc Bonferroni analysis was then performed.

## Results

The mild group (group I): included 35 males and 32 females with a median age of 16 months. The moderate group (group II): included 17 males and 35 females with a median age of 13 months. The severe group (group III): involved 6 males and11 females with a median age of 12 months and the critical group (group IV): included 3 males and 11 females with a median age of 12 months. There were no statistically significant differences between the studied groups regard gender and age. There were statistical differences between the four groups regarding the grades of respiratory distress, MIS –C (multisystem inflammatory syndrome in children), chest CT findings, and need for mechanical ventilation and outcome which reveals higher mortality in both severe and critical groups than the other 2 groups, *chi-square* = *152.015, 150, 157.403, 150,126.43, df* = *9, 3, 9, 3, 3, p* < 0.001, *p* < 0.001, *p* < 0.001, *p* < 0.001, *p* < 0.001 respectively (Table 1).

**Table 1:** Demographic and clinical data in the studied groups

Item	Value	Mild	Moderate	Severe	Critical	*P* value
		***N*** ** = 67**	***N*** ** = 52**	***N*** ** = 17**	***N*** ** = 14**	
**Age (months)**	Median	16	13	12	12	0.953
	IQR	(6–25)	(3.3–42)	(14–16)	(5.5–43.5)	
**Sex**	Male	35(52.2%)	17(32.7%)	6(35.3%)	3(21.4%)	0.059
	Female	32(47.8%)	35(67.3%)	11(64.7%)	11(78.6%)	
**Grades of RD**	G I	33(49.3%)	_	_	_	< 0.001*
	G II	34(50.7%)	32(61.5%)	_	_	
	G III	_	20(38.5%)	7(41.2%)	8(57.1%)	
	G IV	_	_	10(58.8%)	6(42.9%)	
**MIS-C**	No	67(100%)	52(100%)	17(100%)	_	< 0.001*
	Yes	_	_	_	14(100%)	
**Chest CT findings**	CORADS I	_	_	_	_	< 0.001*
	CORADS II	22(32.8%)	_	_	_	
	CORADS III	45(67.2%)	28(53.8%)	_	_	
	CORADS IV	_	24(46.2%)	5 (29.4%)	5 (35.7%)	
	CORADS V	_	_	12(70.6%)	9 (64.3%)	
**Need for MV**	No	67(100%)	52(100%)	_	_	< 0.001*
	Yes	_	_	17(100%)	14(100%)	
**Outcome**	Survived	67(100%)	52(100%)	2(11.8%)	2(14.3%)	< 0.001*
	Died	_	_	15(88.2%)	12(85.7%)	

*IQR* Interquartile range, -*RD* Respiratory distress, -*MIS-C* Multisystem inflammatory syndrome in children, *-CT* Computed tomography, *-CORADS* Covid-19 Reporting and Data System, *MV* Mechanical ventilation

^*^Statistically significant at *p*** < **0.05

Serum ferritin, D-dimer, Presepsin and CXCL 10 were significantly higher in both severe and critical groups more than the other 2 groups, *chi-square* = *72.12, 65.468, 202.914, 309.132, df* = *3,3,3,3, p* < *0.001, p* < 0.001, *p* < *0.001*, *p* < *0.001* respectively, while both serum C3 and C4 were significantly lower in both severe and critical groups than the other 2 groups, *chi-square* = *194.775, 424.014, df* = *3,3, p* < 0.001, *p* < 0.001 respectively (Table 2).

**Table 2:** Inflammatory markers in the studied groups

**Item**		**Mild**	**Moderate**	**Severe**	**Critical**	***P*** ** value**
		***N*** ** = 67**	***N*** ** = 52**	***N*** ** = 17**	***N*** ** = 14**	
**Ferritin (ng/ml)**	Median	291	275	850	1136	< 0.001*
	IQR	(219–350)	(217–324.5)	(604–925)	(937.5–1267.8)	
**D-dimer** **(µg/ml**)	Median	0.8	0.7	8.9	8.9	< 0.001*
	IQR	(0.5–1)	(0.4–2.3)	(4.6–9.8)	(6.3–11.5)	
**C3** **(mg/dl)**	Range	(110–170)	(98–130)	(65–79)	(50–66)	< 0.001*
	Mean ± SD	138.2 ± 18.6	109.5 ± 10.1	71.6 ± 5.3	57.3 ± 5.4	
**C4** **(mg/dl)**	Range	(29–169)	(20–33)	(8–9)	(4–7)	< 0.001*
	Mean ± SD	131.1 ± 28.6	25.1 ± 3.8	8.4 ± 0.4	5.1 ± 1	
**Presepsin** **(ng/l)**	Range	(180–195)	(192–218.6)	(216.4–230)	(221.7–336)	< 0.001*
	Mean ± SD	186.6 ± 4.6	206.4 ± 8.7	224.2 ± 4.7	273.1 ± 36	
**CXCL 10** **(pg/ml)**	Range	(45–59)	(55–95)	(90–300)	(222–500)	< 0.001*
	Mean ± SD	51 ± 4.2	76.1 ± 11.5	198.1 ± 76.9	370.6 ± 93.5	

*C3* Complement 3, *C4* Complement 4, *CXCL 10* Chemokine ligand 10

^*^Statistically significant at *p*** < **0.05

Serum IL- 6 levels showed significant increase in both severe and critical groups when compared with mild and moderate groups, *chi-square* = *302.778, df* = *3, p* < *0.001.*

Concerning the allelic frequencies of the GG, GC and CC genotypes of IL-6 (− 174G/C) were 50.7%, 25.4% and 23.9% in mild case, 40.4%, 36.5% and 23.1% in moderate, zero %, 35.3% and 64.7% in severe case and 14.3%, 7.1% and 78.6% in critical one, respectively. While the allele frequencies of GG, GC and CC genotypes of IL-6 (-572 G/C) were 76.1%, 23.9% and zero in mild case, 36.5%, 42.3% and 21.2 in moderate case, 29.4%, 47.1 and 23.5% in sever and 50%, 35.7% and 14.3 in critical patients. (Table 3).

**Table 3:** Serum IL -6 and Il-6 genotypes allelic frequency in studied groups

**Item**		**Mild**	**Moderate**	**Severe**	**Critical**	***P*** ** value**
		***N*** ** = 67**	***N*** ** = 52**	***N*** ** = 17**	***N*** ** = 14**	
**IL-6** **(pg/L)**	Range	(55–70)	(67–94)	(89–114)	(97–149)	< 0.001*
	Mean ± SD	62 ± 4	83 ± 8	102 ± 8	130 ± 19	
**IL-6 174**	GG	34(50.7%)	21(40.4%)	_	2(14.3%)	< 0.001*
	GC	17(25.4%)	19(36.5%)	6 (35.3%)	1(7.1%)	
	CC	16(23.9%)	12 (23.1%)	11(64.7%)	11(78.6%)	
**IL-6 572**	GG	51(76.1%)	19(36.5%)	5 (29.4%)	7 (50%)	< 0.001*
	GC	16(23.9%)	22(42.3%)	8 (47.1%)	5 (35.7%)	
	CC	0(0%)	11(21.2%)	4 (23.5%)	2 (14.3%)	
**IL-6 597**	GG	34(50.7%)	34(65.4%)	10(58.8%)	8 (57.1%)	0.213
	GA	17(25.4%)	8 (15.4%)	4 (23.5%)	6 (42.9%)	
	AA	16(23.9%)	10(19.2%)	3 (17.6%)	_	

*IL-6* Interleukin 6

^*^Statistically significant at *p*** < **0.05

**Table 4:** Correlations between Serum Presepsin, serum IL-6, CXCL 10 and clinical parameters

Item	Presepsin		IL6		CXCL 10	
	**r**	***p*** ** value**	**r**	***p*** ** value**	**r**	***p*** ** value**
**Mortality**	**0.637**	< 0.001*	**0.624**	< 0.001*	**0.623**	< 0.001*
**Chest CT findings**	**0.757**	< 0.001*	**0.741**	< 0.001*	**0.726**	< 0.001*
**Need for MV**	**0.688**	< 0.001*	**0.683**	< 0.001*	**0.689**	< 0.001*
**Grade of RD**	**0.747**	< 0.001*	**0.762**	< 0.001*	**0.739**	< 0.001*
**MIS-C**	**0.491**	< 0.001*	**0.486**	< 0.001*	**0.495**	< 0.001*

*CT* Computed tomography, *MV* Mechanical ventilation, *RD* Respiratory distress, *MIS-C* Multisystem inflammatory syndrome in children

^*^Statistically significant at *p*** < **0.05

Serum Presepsin, serum IL-6 and CXCL 10 were positively correlated with mortality, chest CT findings, need for MV, grade of RD and MIS-C, *p*<0.001 each (Table 4).

**Fig. 1:**
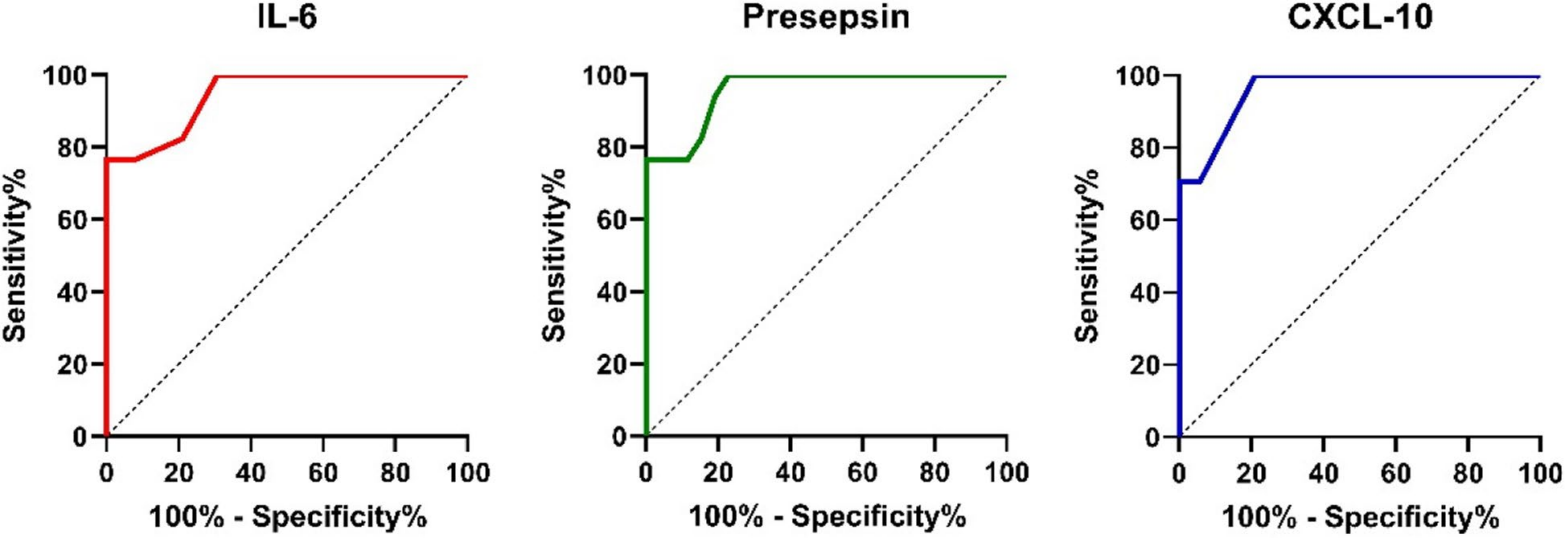
ROC curve analysis of serum IL-6, presepsin and CXCL-10 in severe cases

ROC (Receiver Operating Characteristic) curve revealed a cut off value of > 92 for serum IL-6 with AUC (area under curve) of 0.946 (p < 0.001) in severe cases (sensitivity 76.47%, specificity 92.31%)**.** Concerning serum Presepsin level, ROC curve analysis in severe cases showed a cut off value > 217.7 with AUC of 0.959 (*p* < 0.001) (sensitivity 76.47%, specificity 88.46%), ROC curve analysis in severe cases showed serum CXCL-10 cut off value > 90 with AUC of 0.960 (*p* < 0.001) (sensitivity 70.59%, 94.23%) (Fig. 1).

**Fig. 2:**
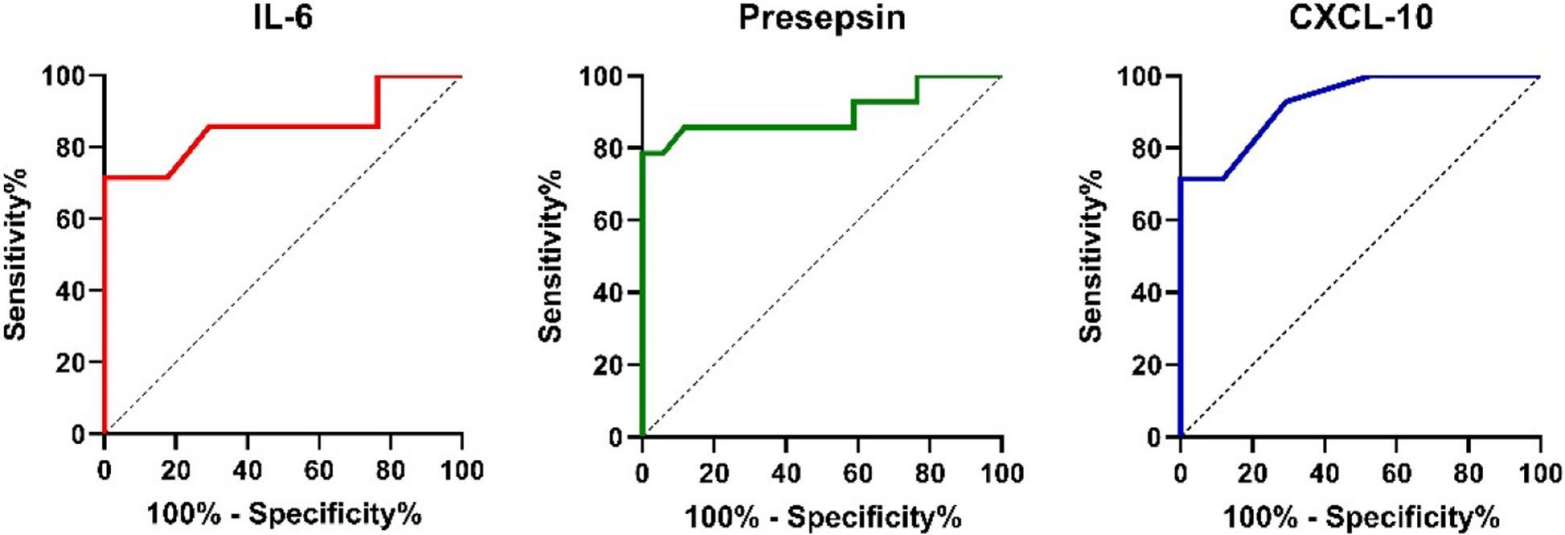
ROC curve analysis of serum IL-6, presepsin and CXCL-10 in critical cases

ROC curve for serum IL-6 in critical cases revealed that cut off value of > 107 with AUC of 0.897 (*p* < 0.001) (sensitivity 71.43%, specificity 82.35%). Regarding serum Presepsin level, ROC curve analysis in critical cases showed serum Presepsin cut off value > 229.8 with AUC of 0.897 (*p* < 0.001) (sensitivity 78.57%, specificity 91.2%). ROC curve analysis for serum CXCL-10 in critical cases revealed a cut off value > 280 with AUC of 0.926 (*p* < 0.001) (sensitivity 71.43%, specificity 88.24%) (Fig. 2).

Regarding genetic analysis, the frequencies of both IL-6 (174) and IL-6 (597) genotypes between severe and mild cases show no significant difference, while IL-6 (572) frequencies show significant difference between mild and severe cases, *p* = 0.011 (Table 5).

**Table 5:** Comparison of the Studied groups as regard genotype and allelic frequency of IL-6 polymorphisms among severe patients

Item	Genotype	Mild	Severe	OR	95% CI	*P* value
		***N*** ** = 67**	***N*** ** = 17**			
**IL-6 174**	GG	34(50.7%)	_	Ref		
	GC	17(25.4%)	6 (35.3%)	NA	NA	NA
	CC	16(23.9%)	11(64.7%)	NA	NA	NA
**IL-6 572**	GG	51 (76.1%)	5 (29.4%)	Ref		
	GC	16(23.9%)	8 (47.1%)	**5.1**	**1.46–17.81**	0.011*
	CC	_	4 (23.5%)	NA	NA	NA
**IL-6 597**	GG	34(50.7%)	10(58.8%)	Ref		
	GA	17(25.4%)	4(23.5%)	0.8	0.22–2.93	0.736
	AA	16(23.9%)	3(17.6%)	0.64	0.15–2.64	0.534

*IL-6* Interleukin 6, *OR* Odds Ratio, *CI* Confidence Interval, *Ref* Reference, *NA* Not Applicable

^*^Statistically significant at *p*** < **0.05

When comparing allelic frequencies between mild and critical cases, only IL-6 (174) and IL-6 572 showed significant difference between the two groups, *p* = 0.003, *p* = 0.022 respectively (Table 6).

**Table 6:** Comparison of the Studied groups as regard genotype and allelic frequency of IL-6 polymorphisms among critical patients

Item	Genotype	Mild	Critical	OR	95% CI	*P* value
		***N*** ** = 67**	***N*** ** = 14**			
**IL-6 174**	GG	34(50.7%)	2(14.3%)	Ref		
	GC	17(25.4%)	1(7.1%)	1	0.09–11.82	1
	CC	16(23.9%)	11(78.6%)	**11.69**	**2.31–59.03**	0.003*
**IL-6 572**	GG	51(76.1%)	7(50%)	Ref	**1.24–16.01**	
	GC	16(23.9)	5(35.7)	**4.46**		0.022*
	CC	_	2(14.3%)	NA	NA	NA
**IL-6 597**	GG	34(50.7)	8(57.1)	Ref		
	GA	17(25.4)	6(42.9)	1.5	0.45–5.02	0.511
	AA	16(23.9)	_	NA	NA	NA

*IL-6* Interleukin 6, *OR* Odds Ratio, *CI* Confidence Interval, *Ref* Reference, *NA* Not Applicable

^*^Statistically significant at *p*** < **0.05

## Discussion

According to our study results, the severe and critical groups had significantly greater levels of IL-6 than the other 2 groups.

This agrees with Chen et al., 2020 who observed that serum levels of IL-6 increased tenfold among critically ill patients than other patients [30].

IL-6 is one of the primary pro-inflammatory mediators involved in the formation of cytokine storms that leads to enhancing vascular permeability and subsequent organ dysfunction. Thus, IL-6 acts as a critical mediator of respiratory failure and multi-organ dysfunction [31]. Cytokine storm is linked to high levels of several pro-inflammatory cytokines like (IL-6), IL-10, IL- 18, (IFN)-y, (TNF- a) as well as inflammatory markers (CXCL-10, presepsin, C3 and C4) [32, 33].

In our study, we found a significant difference in interleukin-6 polymorphisms (IL-6 174) and (IL-6 572) between the four groups. On the contrary, there was no statistically significant variation among the four groups regarding interleukin-6 polymorphisms (IL-6 597). Kerget and Kerget, 2021 found significant difference between NON-MAS (macrophage activation syndrome) and MAS (macrophage activation syndrome) groups as regards IL-6-174G/C polymorphism and this was accompanying with higher IL-6 levels. They also found no significant relation between the severity of covid-19 in adult Turkish patients and the IL-6 597G/C polymorphism [34].

In 2020, Ulhaq and Soraya observed a substantial correlation (*p* = 0.019) between IL-6 174G/C polymorphism and the severity of pneumonia specifically in the Caucasian population [7].

This was in contrast with the finding of Falahi et al., 2022 who demonstrated that no variations in the 174 G > C allele distribution or genotype in the IL-6 gene promoter region between patients with mild and severe COVID-19. They also found no significant variations in the genotype or allele distribution of rs1800797 (-597G/C) in the IL-6 gene promoter region between patients with mild and severe COVID-19 in Iranian population [29]. We found that that IL-6 cut-off value of > 92 pg/ml, area under curve (AUC) of 0.946 for severe cases, *p* value of < 0.001 (sensitivity of 76.47% and specificity of 92.31%) and a cut-off value > 107 pg/ml for critical cases, area under curve (AUC) of 0.857, *p* value of < 0.001 (sensitivity of 71.43% and specificity of 82.35%).

Ganda et al. reported that IL-6 cut-off point had a 93% sensitivity and 90% specificity and it was > 80.97 pg/ml. 0.981 (95% CI), 0.960–1.000) was the AUC [35].

Ismail et al. reported that elevated serum IL-6 levels in patients with COVID-19-infection were related to a variety of outcomes, including severe illness, mechanical ventilation, and acute respiratory distress syndrome, they also concluded that the optimum IL-6 cutoff levels 120.83 pg/ml [36].

Shalaby et al. concluded that the IL-6 value was a good predictive marker for severity (with IL-6 cutoff value > 56) also it can be a predictive marker for COVID-19- infected patients mortality (with a cutoff value > 67) [37]. Zhang et al. concluded that hospital death can be predicted by an IL-6 level more than 37.65 pg/ml (AUC 0.97 [95% CI 0.95–0.99], *P* < 0.001) with a 91.7% sensitivity and a 95.7% specificity [38]. In our study, we found statistical significant difference in IL-6 572 allelic among moderate cases with the most frequent 42.3% for genotype (GC) and allelic among severe cases with the most frequent 47.1% for genotype (GC). Thus, presence of GC genotype of IL-6 572 is considered as a risk factor in both covid-19 patients (moderate and severe).

This is in contrast with Falahi S et al., 2022 who found that no significant difference in IL-6 572 allelic among neither moderate nor severe cases in Iranian adults [29].

We found significant difference in IL-6 174 allelic among critical cases with the most frequent 78.6% for genotype (CC) {Odds Ratio = 11.69}. Thus, presence of GC genotype of IL-6 174 is considered as risk factor in critical covid-19 patients.

This agrees with Verma et al., 2022 who found that in COVID 19 severe patients, C allele and GC genotype of the IL6 gene's -174G/C (rs1800795) polymorphism was greater in them in comparison to mild one (p = 0.009) [39]. Furthermore, our result agrees with Ulhaq ZS and Soraya GV, 2020 who found that individuals who are carrier for C allele of the − 174G / C (rs1800795) polymorphism associated with high IL6 production and severe pneumonia [7].

Our study shows no statistically difference in IL-6 597 allelic among all groups of our patients (mild, moderate, severe, and critical).

This agrees with Falahi S et al., 2022 who found that no statistically significant difference in the genotype and allele frequencies of IL-6 597 polymorphism between the two groups of adult Iranian COVID-19 patients (mild & severe) [29]. Nikhil Kirtipal and Shiv Bharadwaj, 2020 concluded that IL6 polymorphism is essential for understanding the treatment response to COVID-19 in infected humans and for developing population-based therapeutics [40].

We found that both Presepsin and CXCL 10 serum levels were significantly high in both severe and critical groups than the other 2 groups.

Assal et al., 2022 found that Presepsin level was found to be significantly high in non- survivor versus survivor group and strongly correlated with mortality [41]. Lorè et al., 2021 reported that CXCL10 concentration showed significant high level with poor outcome [22].

A previous study by Caldarale et al. found that patients with MIS-C had higher levels of IL-6 and CXCL10 compared to those with COVID-19 [42].

The results of our study's ROC curve showed that Presepsin cut-off value of > 217.7 ng/L in severe cases has an area under curve (AUC) of 0.959, *p* value of < 0.001 (sensitivity of 76.47% and specificity of 88.46%) and the cut-off value of > 229.8 ng/L for critical cases has an area under curve (AUC) of 0.897, *p* value of < 0.001 (sensitivity of 78.57% and specificity of 91.2%).

Fukada et al. and Zaninotto conducted that Presepsin cut-off value of > 250 pg/mL was significant (*p* < 0.05 and *p* < 0.001 respectively) [43, 44].

Farag et al. stated that a cut off value of Presepsin > 330 pg/ml, the corresponding values for specificity, sensitivity, positive predictive value (PPV) and negative predictive value (NPV) were 100%, 60%, 100% and 72.7% [45].

On contrast, Çağlar et al. stated that Presepsin cutoff value of 42.79 pg/ml can predicted severe to critical infection with 64.4% sensitivity and 52.5% specificity [46]. In our study, CXCL 10 has a cut-off value of > 90 pg/ml in severe patients and > 280 pg/ml in critical patients with area under curve (AUC) of 0.960 ND 0.926, *p* value of < 0.001 (sensitivity of 70.59% and 71.43% and specificity of 94.23% and 88.24%. respectively).

Our study showed that severe and critical groups expressed significant lowest levels for both C3 and C4.

This agrees with Zinellu & Mangoni, 2021 who found low serum level of C3 and C4 suggesting more complement activation and product consumption, which is substantially associated with severe illness and higher mortality in COVID-19 patients [47].

Systemic pro-inflammatory state, pro-oxidant condition, and pro-coagulant state with multi-organ affection and higher risk of worse clinical outcome, primarily due to excessive and limitless complement activation [48].

Serum level of C3 and C4 are helpful in diagnosis and monitoring of infectious and immunological complex disorders. C3 is usually reduced due to depletion during infections; however both C3 and C4 levels are reduced in immunological complex illness. Activation of the C3 exacerbates acute respiratory distress syndrome according to a report on COVID- 19 which is closely related to COVID- 19 [49].

According to the Cox regression analysis, there was a reverse correlation between low C3 concentration and worse condition in patients with COVID-19. Multiple analyses generated supported this evidence. Moreover, previous research indicates that low level of C3 may increase possibility of death [50].

We also found that D-dimer levels were significantly higher in both severe and critical groups compared to the other 2 groups.

D-dimer is the result of fibrinolytic destruction of fibrin and increased levels means presence of hypercoagulable state and secondary fibrinolysis, which is valuable for diagnosis of thrombotic disorders [51].

Seventy one percent of Patients with COVID-19 died from DIC due to hypercoagulable state [52].

In our study, we found higher levels in serum ferritin in both severe and critical groups when compared with the other 2 groups.

This agrees with Deng et al., 2021 who found high-ferritin level on admission, and it was associated with higher incidence of mortality [53].

Ferritin is a protein that increases in response to different inflammatory states in addition to malignancies, iron overload, and liver or kidney diseases [54].

There were significant differences between our four groups regarding grades of respiratory distress, MIS- C, chest CT findings, need for mechanical ventilation and outcome which revealed higher mortality in both severe and critical groups than the other 2 groups.

Kim et al., 2020 also found more severe manifestations and respiratory distress in both severe and critical patients [55].

Chung et al., 2020 detected more advanced chest CT changes in severe and critical patients. Feldstein et al., 2021 also demonstrated features of MIS-C which develops with worsening of the general condition of patients and thus, increase the need for mechanical ventilation in these 2 groups (severe and critical) [56, 57].

Some limitations of the present study were (1) it was a single-center and observational study; (2) the sample size was small; (3) the markers serum levels were measured only once.

## Conclusions

Our study can conclude that children whom expressed GC genotypes of IL6 (-572G > C) polymorphism are at a considerably higher risk of developing a severe disease. This risk is significantly larger in the severe group of children than in children in critical condition who have GC genotypes of IL6 (-174 G > C) polymorphism. While IL6 (-597G > A) polymorphism has no role in COVID 19 severity in child.

## Data Availability

The datasets used and/or analysed during the current study are available from the corresponding author on reasonable request.

## Abbreviations

COVID-19: Corona Virus Disease-2019
INF-y: Interferon gamma
TNF- α: Tumor necrosis factor
IL6: Interleukin 6
CXCL10: Chemokine ligand 10
C3: Complement 3
C4: Complement 4
SNPs: Single nucleotide polymorphisms
CBC: Complete blood count
CRP: C reactive protein
ELFA: Enzyme Linked Fluorescent Assay
PCR-REFLP: Polymerase chain reaction-restriction fragment length polymorphism
MIS-C: Multisystem Inflammatory Syndrome in Children
CT: Computed tomography
MV: Mechanical ventilation
RD: Respiratory distress
CORADS: Covid-19 Reporting And Data System
ROC: Receiver operating characteristic
AUC: Area under the curve
OR: Odds Ratio
CI: Confidence Interval
Ref: Reference
NA: Not Applicable
SARS- CoV-2: Severe acute respiratory syndrome coronavirus 2
BMI: Body mass index
MPP: Mycoplasma pneumoniae
PPV: Positive predictive value
NPV: Negative predictive value
MAS: Macrophage activation syndrome
LDH: Lactate dehydrogenase
ALT: Alanine aminotransferase
AST: Aspartate aminotransferase
